# Analysis of malignant hyperthermia results in Padua and Siena

**DOI:** 10.1186/1471-2253-14-S1-A4

**Published:** 2014-08-18

**Authors:** Vincenzo Tegazzin, Alfredo Orrico, Lucia Galli, Marina Fanin, Daniela Rossi

**Affiliations:** 1 Department of Anesthesia, University of Padua, Padua, 35100, Italy; 2 Molecular Medicine Section, Department of Molecular and Developmental Medicine, University of Siena, via A. Moro 2, Siena, 53100, Italy; 3 Neurological Science, University of Padua, Padua, 35100, Italy

## Background

Malignant Hyperthermia is pharmacogenetic disease triggered by some anaesthetics such as halogenated gases and non depolarizing muscle relaxant. The diagnosis of malignant hyperthermia susceptibility may be done by 1) relevant clinical signs observed during the crisis, 2) the in vitro contracture test (IVCT) with caffeine-halothane according to the European and the American Malignant Hyperthermia Group protocols and/or 3) the search for mutations of RYR 1 and CACNA1S genes.

The IVCT identified three categories of patients: a) MHS (malignant hyperthermia susceptible), b) MHE (malignant hyperthermia equivocal) and c) MHN (non susceptible).

A gene mutation may be defined as diagnostic only if it can be defined as “causative mutations” capable to alter the protein function in a cell culture test. The patients carrying “no causative” mutations must carry out the IVCT to confirm a susceptibility condition.

Usually, the first investigated patients, the probands, have had an abnormal reactions during trigger anaesthesia such as early signs (inappropriately elevated CO_2_, inappropriate tachycardia and/or arrhythmia and muscle signs like masseter spasm or generalized muscle rigidity) or late signs (rapid increase in core body temperature, high value of CK etc). If the anaesthesia report has been well done it is possible to attribute a Larach Clinical Grading Scale that classifying the reactions as” almost certain” (more than 50 scores) or very rare “almost never” with 0 scores.

The susceptible probands may be also genetically studied and the relatives warned of the genetic risk of an autosomal dominant trait. The problems for the physicians may be relevant when the probands have a raw score range from 20 to 34 or more, and the IVCT results is negative (MHN).

Although negative patients may be happy to result negative, they remain worried regarding anaesthesia own and of their relatives.

The worry for General Anesthesia (GA) does not disappear with a negative test and it may be very difficult explaining that other possible causes may be involved in MH like reactions. In some MHN probands it may be easier to find an explanation, as it is possible an alternative diagnosis or because…. the not clear or wrong anaesthetic report but what about the others. In some of similar cases, we decide to perform a genetic analysis and in one patient MHN patient we found a “no causative mutation”. At this point the question is 1) can be assure the MHN patients regarding GA with trigger agents? 2) Are there other possible investigations to give more assurance to such patients? 3) genetic testing should be prescribed also in the MHN probands?

We present the data of our MH laboratories highlighting the IVCT and genetic results obtained on our probands and the suggestions and information that we give to the MHN probands as well as some questions to the auditorium.

